# New Strategies for Rehabilitation and Pharmacological Treatment of Fatigue Syndrome in Multiple Sclerosis

**DOI:** 10.3390/jcm9113592

**Published:** 2020-11-07

**Authors:** Ewa Zielińska-Nowak, Lidia Włodarczyk, Joanna Kostka, Elżbieta Miller

**Affiliations:** 1 Department of Neurological Rehabilitation, Medical University of Lodz, Milionowa 14, 90-001 Lodz, Poland; ewa.zielinska@umed.lodz.pl; 2 Department of Occupational Diseases and Environmental Health, Nofer Institute of Occupational Medicine, 91-348 Lodz, Poland; lidia.monika.wlodarczyk@gmail.com; 3 Department of Gerontology, Medical University of Lodz, Milionowa 14, 90-001 Lodz, Poland; joanna.kostka@umed.lodz.pl

**Keywords:** multiple sclerosis, fatigue, pharmacological treatment, rehabilitation, physical activity, aerobic training, functional electrical stimulation, non-invasive brain stimulation

## Abstract

Multiple sclerosis (MS) is the most common autoimmune disease of the central nervous system (CNS), with an inflammatory demyelinating basis and a progressive course. The course of the disease is very diverse and unpredictable. Patients face many problems on a daily basis, such as problems with vision; sensory, balance, and gait disturbances; pain; muscle weakness; spasticity; tremor; urinary and fecal disorders; depression; and rapidly growing fatigue, which significantly influences quality of life among MS patients. Excessive fatigue occurs in most MS patients in all stages of this disease and is named MS-related fatigue. The crucial issue is the lack of effective treatment; therefore, this review focuses not only on the most common treatment methods, but also on additional novel therapies such as whole-body cryotherapy (WBC), functional electrical stimulation (FES), and non-invasive brain stimulation (NIBS). We also highlight the advantages and disadvantages of the most popular clinical scales used to measure fatigue. The entire understanding of the origins of MS-related fatigue may lead to the development of more effective strategies that can improve quality of life among MS patients. A literature search was performed using MEDLINE, EMBASE, and PEDro databases.

## 1. Introduction

Multiple sclerosis (MS) is the most common autoimmune disease of the central nervous system, with an inflammatory demyelinating and progressive course of the disease in humans (usually onset is at 20–40 years of age). The etiology of this disease is still not fully understood [1]. In Europe, the total estimated prevalence rate for MS is 83 per 100,000, with higher rates for women than men [2].

The course of the disease varies considerably depending on the patient, with the differences relating to the rate of the disease development, age of onset, and the number and severity of symptoms. Taking into account the clinical course and the progression of symptoms, four main disease courses have been specified: relapsing–remitting (RRMS), secondary progressive (SPMS), primary progressive (PPMS), and progressive relapsing MS (PRMS).

The clinical picture in MS is very diverse and variable and the symptomatology depends on the localization of damage in the central nervous system. The onset of the disease is usually monosymptomatic, and over time it turns into a wide range of coexisting symptoms, such as paralysis, ataxia, spasticity, and fatigue syndrome (FS) [1].

The problem of fatigue affects the vast majority (up to 75%) of patients with MS and is reported as one of the most burdensome symptoms [3].

The aim of this study is to show the effectiveness of various methods, both pharmacological and non-pharmacological, in the treatment of chronic fatigue in patients with MS. An attempt to present not only standard methods, but also new, modern methods that can support the effectiveness of therapy in this group of patients has been made.

### 1.1. Fatigue in Multiple Sclerosis

Fatigue is defined as an overwhelming feeling of exhaustion and lack of energy. Patients have the impression that the effort that they have to put into performing a given task is disproportionately high, which as a consequence leads to the avoidance of physical activity [4]. Fatigue is a subjective symptom, therefore it can be confused with a feeling of general weakness or depression [4]. Patients should be carefully examined physically and psychologically, so as not to confuse symptoms of FS with depression or other ailments, as these can require separate treatments.

MS patients with heat sensitivity can experience fatigue as a symptom related to heat, which is identified by the occurrence of a conduction block in Ranvier’s nodes (Uhthoff’s phenomenon) [5]. Factors such as depression, certain medications (opioids, long-term benzodiazepines, sedatives, painkillers, anticonvulsants, muscle relaxants, or pressure-reducing medications), alcohol or nicotine, sleep disturbances, infections, and fever also exacerbate fatigue [6,7,8]. Moreover, fatigue usually increases in the afternoon and may be aggravated by stress or even slight physical or mental exertion [9,10].

MS-related fatigue differs from physiological fatigue (tiredness) by being more intense, which is not reduced by sleep or rest, and also by lasting a longer time [11]. A higher prevalence of severe fatigue is observed in progressive relapsing MS compared with relapsing–remitting and primary progressive MS [12]. The symptom of fatigue may herald a relapse and may occur even several weeks or months before it, however it may also persist for a long time and not correlate with relapse or remission [4]. 

MS-related fatigue can be divided into primary fatigue, which is correlated with demyelination and axonal loss in the CNS, and secondary fatigue, which occurs as a result of MS-related complications, such as sleep disorders, reduced activity, and depression [13]. Physical fatigue refers to bodily exhaustion after performing a physically engaging task, whereas cognitive fatigue is associated with mental exhaustion [14].

Despite the fact that many studies have already been performed, it is still not possible to clearly establish the underlying causes of FS. Among many hypotheses, the most common are disorders of the immune system or sequelae from CNS damage. Specific causes include proinflammatory cytokines (increased TNF-α mRNA expression, TNF-α levels, and interferon-γ levels), endocrine influences (decrease in DHEA concentration), axonal loss or increased volumes, and patterns of cerebral activation [15]. 

### 1.2. Fatigue Syndrome Diagnosis

More than 30 questionnaires have already been created to diagnose this syndrome, but due to the high subjectivity it is difficult to indicate one universal scale. The most widely used scales are the fatigue severity scale (FSS), because it is short (nine questions) and examines several aspects of fatigue; the modified fatigue impact scale (MFIS), comprising 21 questions; the fatigue descriptive scale (FDS); and the visual analogue scale for fatigue (VAS-F) [16]. It is common knowledge that self-report questionnaires are influenced by a variety of factors, not only symptoms of MS, but also social, environmental and emotional factors. Consequently, this estimation is completely subjective. Despite these limitations, both scales (FSS and MFIS) are most commonly used in clinical trials, and therefore their outcomes can be easily compared by scientists. 

Tellez et al. compared these two scales in a study on 354 individuals (231 MS patients and 123 healthy controls) and suggested that both scales provide similar measurements, although cognition and psychosocial functions are more thoroughly measured by the MFIS [17]. Objective assessment of the FS is limited to the observation of a patient while performing psychological and motor exercises, due to the fact that fatigue is characterized by a decrease in strength, concentration, and speed of tasks performance over time, with very large differences in the results of these parameters at the beginning of the task and at the end. Another phenomenon is the observation made during the performance of cognitive tasks, characterized by a decrease in the ability to react and the accuracy of task performance over time during the therapy. Moreover, the key problem in fatigue diagnosis is distinguishing fatigue from depression, cognitive impairment, and sleep disorders. Considering MS-related fatigue is a complex problem, we should use a variety of tools to make a proper diagnosis [18,19,20].

## 2. Methods

The literature search was performed using MEDLINE, EMBASE, and PEDro databases. In total, 57 articles were analyzed, including 46 original research papers and 11 reviews (meta-analyses, systematic reviews, literature reviews). At the beginning we included articles from the last 10 years, however in order to broaden the perspective the search was extended until the year 2000. All articles that were included covered strategies for rehabilitation and pharmacological treatment of MS-related fatigue and were published in English. Search terms included fatigue therapy, multiple sclerosis, pharmacological treatment, exercise, physical activity, and physical agents. We excluded articles published before the year 2000 in languages other than English and articles that did not mention pharmacological treatment or rehabilitation of MS-related fatigue. No restrictions were set for the type of MS, disability level, or severity of fatigue or disease. Two authors independently searched databases for articles on pharmacological agents and two searched for non-pharmacological therapeutic agents that affect fatigue in MS patients.

## 3. Pharmacological Treatment of the Fatigue Syndrome

Treatment of the FS is extremely difficult due to the lack of thorough understanding of its etiology. The first step in fatigue treatment is ruling out depression and factors that may worsen fatigue, such as sleep disturbances, overheating, stimulants, and pain. The necessity to take certain medications should also be reconsidered (such as benzodiazepines, opiates, codeine-containing drugs, myorelaxants, and anti-anxiety and anti-depressant medications). The standard neurological treatment for MS is focused on reducing the frequency of clinical relapses and new lesion formations. Amantadine, paroxetine, modafinil, and 4-aminopyridine, often taken together with antidepressants, are recommended in neurological fatigue treatment routines [21].

4-Aminopyridine (4-AP) is potent inhibitor of voltage-gated potassium channels (Kv). Studies have shown that 4-AP can improve conduction of action potentials in demyelinated nerve fibers, thereby increasing the release of neurotransmitters in synapses and at the neuromuscular junction [22]. Rossini et al. divided patients treated with 4-aminopyridine into two groups, depending on the concentration of the serum, and observed a positive effect on fatigue in individuals with high serum concentrations (>30 ng/mL) in comparison to the placebo group [23]. Another drug is amantadine, which is an antiviral agent. The mechanism of its action for treating fatigue among MS patients is unclear, but it may be related to antiviral activity, immune-mediated activity, or an amphetamine-like action [24]. In a blinded, randomized, controlled trial, 60 adult patients with relapsing–remitting MS received 1 month of treatment with amantadine (200 mg daily), acetyl-L-carnitine (2 g daily), modafinil (200 mg daily), or placebo. The outcome was measured using the MFIS scale, and after the treatment period there was a significant difference in contrast analysis between patients on amantadine and the placebo group. No changes or only slight changes were found in the group taking modafinil and acetyl-L-carnitine [25]. Möller et al. also do no not support using modafinil as a treatment for fatigue in MS [26]. A similar clarity applies in Stankoff’s randomized, placebo-controlled, double-blind study with modafinil vs. placebo, where in both placebo-treated and modafinil-treated groups MFIS scores improved but no significant difference was detected between the two groups [27]. Otherwise, in a small study by Lange et al., fatigue measured by FSS improved significantly in the modafinil group and remained better than in the placebo group after 8 weeks of treatment [28]. To compare the efficacy of acetyl-L-carnitine (ALCAR) with that of amantadine, one of the drugs most widely used to treat MS-related fatigue, 36 MS patients presenting fatigue were enrolled in a randomized, double-blind, crossover study [29]. Statistical analysis showed significant effects of ALCAR compared with amantadine for the FSS (*p* = 0.039). 

In this report, the last analyzed drug is paroxetine, which is an antidepressant from the selective serotonin reuptake inhibitors (SSRIs) group. Although paroxetine is mainly prescribed for depression, it has also proven to be effective in decreasing fatigue (as measured by the MFIS scale) [30]. According to a meta-analysis prepared by Tsou et al., only paroxetine improved fatigue, but there is a lack of evidence for amantadine, modafinil, and methylphenidate as treatments for FS [31]. In contrast, the meta-analysis performed by Yang at al. indicated that amantadine, not modafinil, was effective in treating MS-related fatigue. They concluded that current data could not answer whether treatment of MS fatigue using aspirin or 4-aminopyridine is beneficial [32]. 

As seen above, many drugs have already been tested in the treatment of MS fatigue, however the evidence supporting their effectiveness is uncertain. Studies involving small sample sizes have had conflicting results. Therefore, more studies should be performed to create evidence-based and personalized treatment options for patients affected by MS-related fatigue. We are certainly looking forward to the results of a multicenter trial of three commonly used medications for the treatment of MS-related fatigue (amantadine, modafinil, methylphenidate) versus placebo in fatigued subjects with MS [33]. Table 1 summarizes the standard neurological drugs recommended for MS-related fatigue and the main findings from clinical studies (Table 1).

## 4. Non-Pharmacological Treatment of Fatigue Syndrome

MS is long-lasting disease with clinical progression of irreversible symptoms, for which conventional therapy is often not effective. It seems that new therapies should be more targeted to one particular symptom. In 2014, the American Academy of Neurology published a comprehensive literature review and evidence-based practice guidelines for complementary and alternative medical therapies (CAM) for MS [35]. Several oral therapies, such as cannabis, ginkgo biloba, magnetic field therapy, and reflexology, were shown to be potentially effective for treating MS-related fatigue, disability, and for improving quality of life.

### 4.1. Physical Rehabilitation

The least invasive method of treatment is physiotherapy. In one study, it has been shown that the implementation of an appropriate training plan largely based on aerobic exercise reduces fatigue by about 40–50%, however the exercise program should be individually adjusted, taking into account all possible symptoms of patients with MS and co-existing diseases [36]. Rehabilitation of patients with MS is a particularly demanding task due to the wide spectrum of symptoms and the inability to predict the course of the disease. 

According to the meta-analysis of different types of fatigue management in MS patients, interventions based on rehabilitation may have even stronger and more significant effects on reducing fatigue than medication [37].

#### 4.1.1. Physical Activity and Exercise Therapy

Current studies support the statement that training programs in MS cause positive effects, especially for those with mild and moderate disability levels. There is some evidence that physically active MS persons are characterized by better results in fatigue scales than non-exercisers [38]. According to the Cochrane review based on 36 studies involving 1603 people with MS, exercise interventions (particularly endurance, mixed, or “other” training) are safe and may be effective in reducing fatigue symptoms. However, the authors note that in the future, high-quality research is needed to confirm the effectiveness of exercise therapy [39].

There are some potential mechanisms that may explain the beneficial effects of physical activity and exercise on both primary and secondary fatigue in people with MS. In the case of primary fatigue, attention is paid to changes in the CNS under the influence of regular exercise (decreased neurodegeneration, improved synaptic plasticity and neurogenesis through increased BDNF level), immunologic changes (reduction of inflammation), and neuroendocrine changes (through normalization of hypothalamic–pituitary–adrenal axis dysfunction). Many of the above-mentioned effects of exercise (especially aerobic training) can also positively influence secondary fatigue. It is known that regular physical activity induces benefits, such as improvements in quality of sleep and mood, reduction of depression symptoms, and improvement of aerobic capacity caused by positive changes in cardiovascular and locomotor systems. These improvements positively influence motor functions and reduce energy expenditure during everyday activities [40]. 

Despite the evidence of the safety and beneficial effects (including fatigue reduction) of physical activity in MS, people with MS are less active than the healthy controls [41]. Meanwhile, deconditioning related to inactivity has many negative consequences, including deterioration of functional fitness and intensification of fatigue symptoms. Due to the wide range of symptoms and the severity of the disease, recent recommendations for physical activity for MS patients include different exercise strategies and physical activity recommendations, depending on the degree of disability (based on expanded disability status scale (EDSS) ranges) [42]. These recommendations include many different forms of physical activity and exercise that may be performed by MS patients, such as aerobic activity (e.g., cycle ergometry, treadmill or overground walking, rowing, jogging, aquatic activities), resistance training, flexibility exercises, neuromotor training (including pilates, dance, yoga, tai chi, hippotherapy, exercise with virtual reality), and breathing exercises. The intensity of the activity, the form of exercise, and the training volume, as mentioned above, depend on the severity of the disease. However, even the most vulnerable patients (EDSS 9.0—inability to perform most activities of daily living) should be as active as possible. On the other hand, even intense exercise such as running or cycling is allowed in people with SM with mild impairment.

Most often moderate exercise intensity is recommended for MS individuals, however single studies show that high-intensity resistance training [43] and high-intensity aerobic training [44] are safe and may lead to even better improvements in many aspects of functioning, including measures of fatigue in MS individuals. Actually, there are some studies that have presented reductions of fatigue in FSS or MFIS scales after applying aerobic exercise programs [45,46,47].

Although physical activity is recommended in MS, it is known that exercise increases the metabolic rate, which is connected with heat generation and an increase of body temperature, which may not be suitable for heat-sensitive patients with MS. Such patients may benefit from training in a cooled room. Devasahayam et al. studied a vigorous aerobic walking program conducted in a room cooled to 16 °C using a bodyweight-supported treadmill (BWST) for people with moderate to severe MS-related disability, which resulted in a reduction of the fatigue symptom [48].

Some studies indicate a positive influence of aquatic exercises on MS-related fatigue [49,50,51]. This method also provides the relief and ideal resistance for light strengthening exercises. The additional benefit of exercising in water is a sense of security in case of losing balance and the risk of falling. Additionally, thanks to the aquatic environment being at the right temperature, the effect of muscle relaxation appears. It was also proven that patients who practiced yoga under the supervision of a qualified instructor had lower MFIS scores after 8 weeks of three yoga sessions per week [52]. 

Music therapy may also be an interesting way of diversifying the training. This therapy stimulates patients to move and allows them to keep their mind off of the disease for a while, forgetting about the pain and limitations and simply performing movements to the rhythm of music. Dance training may have positive effects on fatigue, cognitive performance during a task, and motor functions [53].

Fatigue in MS is associated with functional performance. In the study by Garg et al., MS participants with higher levels of fatigue were characterized by greater impairment of both performance-based and self-reported functional mobility [54]. Taking this into account, the rehabilitation program for this group of patients should be focused on functional deficits. In this area Bobath, Frenkel exercises or Proprioceptive Neuromuscular Facilitation (PNF) approaches are often used for patients with MS [55]. Table 2 presents research articles on different types of physical activity performed by patients with MS-related fatigue (Table 2).

#### 4.1.2. Physical Agents

In order to strengthen the effects of physical rehabilitation, it is worth using therapies involving physical agents. Due to the negative influence of high temperature on nerve conduction and fatigue in MS patients, any procedures involving significant increases of body temperature are not recommended. 

As has been previously reported in the literature, treatments with cold agents are widely used in this group of patients. For instance, applying local cryotherapy to the shoulder area reduces intention or systemic tremor, which has a beneficial effect on the patient’s functional state [59]. Single studies indicate that treatment with cold may have positive effects for both the body and the psyche, and that the improvement in well-being can be particularly observed in people suffering from depression [60]. It is worth paying attention to the possibility of using whole-body cryostimulation (WBC) to reduce fatigue. In the study by Miller et al., after ten sessions of WBC (one exposure per day at −110 °C or lower), patients reported improvements in functional status and feeling of fatigue [61]. Lowering the body temperature with a cooling garment also seems to have a similar positive influence on fatigue [62,63]. Even a single session with a Rehband cooling garment caused many positive changes for MS patients, including subjective improvements in fatigue [64]. The authors of the above-mentioned articles did not report any side effects for the applied treatments [61,62,63,64]. Patients were excluded from the whole-body cryotherapy study if they had any of the following contraindications: antihypertensive or vasoactive medications or diuretics within the previous month; or any other significant medical diagnoses, including thyroid, hypothalamic or cardiovascular disease, circulatory or breathing insufficiency, clotting, embolisms, inflammation of blood vessels, open wounds, ulcers, serious cognitive disturbances, fever, addictions, claustrophobia, or over-sensitivity to cold [61]. Table 3 presents clinical studies on cold therapies in patients with MS-related fatigue.

The influence of pulsed magnetic field therapy (PMFT) has also been evaluated among this group of patients and it seems that it might be helpful in alleviating fatigue. In the study performed by Lappin et al., a daily exposure to a small, portable pulsing electromagnetic field generator caused improvements in fatigue and overall quality of life [65]. The significant difference in MFIS outcomes was also noted after 12 weeks of using BEMER magnetic field treatment for 8 min, twice daily [66]. Although some studies indicate slight positive impacts of PMFT on fatigue, their outcomes were not statistically significant [67,68]. The authors of the above-mentioned articles did not report any side effects for the applied therapies. The most common exclusion criteria were exacerbation of MS, pregnancy, pacemaker, serious or chronic diseases, psychiatric disorders, and epilepsy [65,66,67,68]. Table 4 presents the influence of magnetic field therapy on MS-related fatigue (Table 4).

Functional electrical stimulation (FES) is the next physical agent that is being investigated as a potential treatment of fatigue in MS patients [69,70,71]. 

In the study by Chang et al., 8 weeks of quadriceps muscle surface FES training for individuals with MS led to significantly reduced fatigue. In addition, a very interesting application of FES is FES connected with cycling [70]. During training, the electrodes are placed on the muscles and electrostimulation supports the movements of the muscles that are engaged during cycling. After 24 weeks of FES cycling training, the authors pointed out the benefits of FES cycling exercise on symptoms of fatigue, cognition, and pain [71]. Nevertheless, it should be noted that these studies were conducted in small groups of patients, hence future research should further develop and confirm these initial findings. In the presented articles, the following contraindications to FES have been mentioned: skin lesions; cancerous cells at the site of electrode placement; the presence of a demand-type pacemaker, defibrillator, or any electrical or metallic implant [69]; history of osteoporosis; other musculoskeletal disorders [70]; epilepsy; unstable fractures; pregnancy [71]. No side effects have been reported. Table 5 highlights the potential value of FES in the treatment of MS-related fatigue (Table 5).

### 4.2. New Therapies: Non-Invasive Brain Stimulation

Non-invasive brain stimulation (NIBS) is a novel neuromodulatory method that has shown promising treatment effects on several neurological disorders, such as sequelae of stroke and chronic pain. The evaluation of NIBS treatments, such as transcranial direct current stimulation (tDCS), transcranial magnetic stimulation (TMS), transcranial random noise stimulation (tRNS), transcranial alternating current stimulation (tACS), cranial electrotherapy stimulation (CES), and reduced impedance non-invasive cortical electrostimulation (RINCE), has shown that tDCS is a safe and effective method of treating MS-related fatigue. The reduction of fatigue in the analyzed study was statistically significant both after the last stimulation and also after a long period compared to sham stimulation. There were no significant changes observed for TMS and tRNS [72]. A recent study by Chalah et al. showed that bifrontal tDCS seems to modulate fatigue in patients with MS. Eleven fatigued MS patients randomly received two blocks (active and sham tDCS) of five consecutive daily sessions of bifrontal tDCS (anode and cathode over the left and right prefrontal cortices, respectively) in a crossover manner, separated by a 3-week washout interval. Active but not sham tDCS resulted in a significant improvement of fatigue at day 5 (*p* < 0.05), an effect that seems to last at least 1 week following the stimulation (*p* = 0.05) [73]. Similar results were observed by Canchelli et al. [74]. They have recruited ten patients with MS-related fatigue, who received 5-day transcranial direct current stimulation (tDCS) in a randomized, double-blind, sham-controlled, crossover study, with MFIS score reduction at the end of the treatment as the primary outcome. Likewise, in a study performed by Tecchio et al., similar results were obtained—anodal tDCS over bilateral somatosensory areas was able to reduce fatigue in mildly disabled MS patients [75]. 

Although those studies are based on a small sample of participants, the findings support the concept that interventions modifying the sensorimotor network activity balances could be suitable non-pharmacological treatments for MS-related fatigue. The results of these studies on the effectiveness of NIBS are listed in Table 6.

Another interesting possibility is deep transcranial magnetic stimulation (rTMS). Gaede et al. reported on a positive influence of 18 consecutive deep brain H-coil repetitive rTMS sessions over 6 weeks. The authors drew attention to the significant median FSS decrease of 1.0 point (95% CI (0.45, 1.65)), which was sustained during follow-up [77].

**Figure 1:**
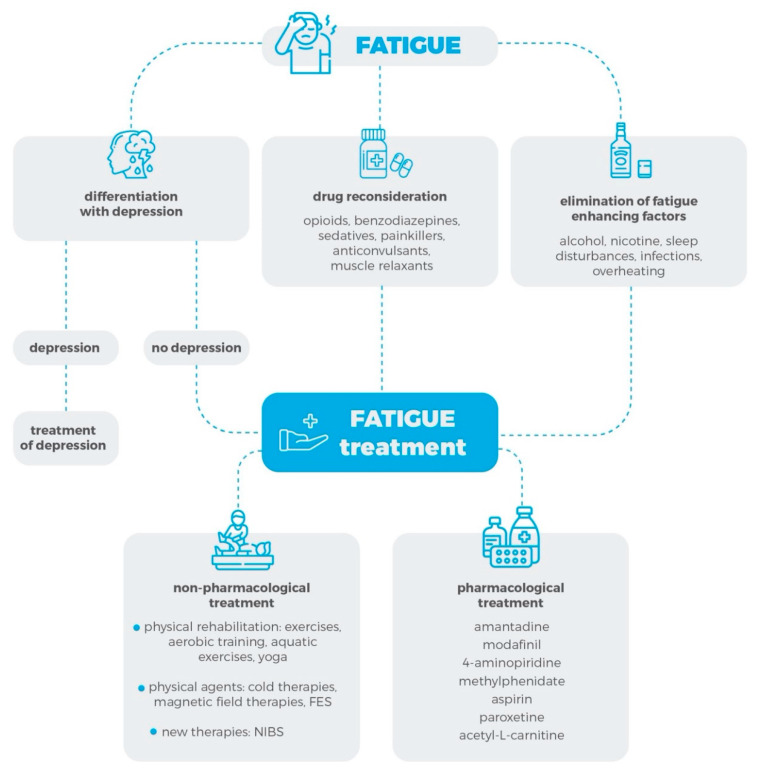
A protocol for the management of fatigue in MS patients, as presented in this review. Abbreviations: FES: Functional Electrical Stimulation; NIBS: Non-Invasive Brain Stimulation. This figure was designed using resources from Flaticon.com.

Figure 1 illustrates the approach to the treatment of patients with MS-related fatigue, which is presented in this review (Figure 1).

## 5. Future Research and Directions

Due to the fact that fatigue in MS patients is a complex clinical problem, future studies should look for not only precise diagnoses of depression and sleepiness, but also complex estimation of additional diseases that can influence fatigue. Currently, there are several techniques used to measure additional factors that can contribute to fatigue in MS. The most important are positron emission tomography (PET) and magnetic resonance imaging (MRI), brief repeatable battery of neuropsychological tests, and peripheral factors such as electromyography [78]. From our point of view, future research studies on MS-related fatigue should be more concentrated on psychological examination to distinguish fatigue from depression. It is very important to include in our thinking about fatigue the decreased physical and mental performance, which can lead to changes in psychological and peripheral factors. However, these changes depend not only on the kind of task that is performed and the environmental conditions, but also on the disease status [79].

## 6. Conclusions

When establishing a treatment plan for MS patients, particular attention should be paid to the thorough diagnosis of the fatigue syndrome, because its occurrence requires a different approach. This review presents a wide range of different therapeutic possibilities that might have a positive impact on MS-related fatigue. However, currently there is not a single versatile and fully effective treatment for this symptom. Therefore, a combination of pharmacological and non-pharmacological therapies, which were discussed in this review, is recommended. It is also worth considering trying new treatment possibilities, such as non-invasive brain stimulation. The aforementioned methods should not be omitted in the treatment plan, although more research should be done in the field of alternative treatment methods, as their use seems to be beneficial without causing significant side effects. Future research should have standardized research protocols and use the same scales in order to present more transparent and unambiguous results.

## Figures and Tables

**Table 1:** Neurological standards for the pharmacological treatment of fatigue.

Study, Year, Reference	Study Design	Specific Treatment	Control Group	Fatigue Outcome Measures	Main Findings
Rossini et al., 2001 [23]	Randomized, controlled trial, *n* = 54, 6 weeks	4-AP	Placebo	FSS	Changes in fatigue scores, EDSS, and cognitive functions were not significantly different between 4-AP and placebo. When patients treated with 4-AP were divided into two groups according to the serum level of 4-AP, a significant effect on fatigue compared with placebo was observed in the “high level” (>30 ng/mL) group (*p=* 0.05).
Ledinek et al., 2013 [25]	Randomized, controlled trial, *n* = 60, 1 month	Amantadine, modafinil, and ALCAR	Placebo	MFIS	Significantly lower mean MFIS score after one month in patients on amantadine compared to placebo (mean difference = 17.3, *p* = 0.001). There was also a trend of lower MFIS scores in ALCAR group in comparison to placebo (mean difference = 12.4, *p* = 0.05, with Keppel-corrected alpha of 0.046).
Möller et al., 2011 [26]	Randomized, controlled trial, *n* = 121, 8 weeks	Modafinil	Placebo	FSS, MFIS	Both treatment groups showed improvements over time. While mean FSS at 8 weeks showed a trend difference between groups in the intention-to-treat analysis, the primary endpoint was not met. Assessment of cognitive impairment by SDMT and PASAT showed contradictory results. All other secondary endpoints were not met.
Stankoff et al., 2005 [27]	Randomized, controlled, double-blind study *n* = 115, 35 days	Modafinil	Placebo	MFIS	The mean MFIS score at baseline was 63 ± 9 in the placebo group and 63 ± 10 in the modafinil group. MFIS scores improved between day 0 and day 35 in both placebo-treated and modafinil-treated groups, but no significant difference was detected between the two groups.
Lange R. et al. 2009 [28]	Double-blind, placebo-controlled study, *n* = 21, 8 weeks	Modafinil	Placebo	FSS	After the first drug ingestion, fatigue measured by the FSS improved significantly in the modafinil group and remained better than in the control group after 8 weeks of treatment. Compared to baseline, FSS was lower after 3 h (*p* = 0.025) and 8 weeks P = 0.01) within the modafinil group.
Tomassini et al., 2004 [29]	Randomized, double-blind, crossover trial, *n* = 36, 3 months	ALCAR	Amantadine	FSS FIS	Statistical analysis showed significant effects of ALCAR compared with amantadine for the FSS (*p* = 0.039).
Ehde et al., 2008 [30]	Randomized, controlled trial, *n* = 42, 12 weeks	Paroxetine	Placebo	MFIS	Treatment participants improved more than controls on the psychosocial subscale of the MFIS (*p* = 0.02) In the treatment group, the 11 participants who responded were compared to the 9 participants who dropped out or who did not respond. Responders had lower MFIS scores (50.5 vs. 64.6; *p* = 0.027), with the most notable MFIS subscore effect on the cognitive scale (21.2 vs. 33.0; *p* = 0.006).
Tsou A et al., 2019 [31]	Meta-analysis of RCTs, *n* = 45 trials	4-AP, amantadine, modafinil, aspirin, paroxetine	Placebo		Only 1 pharmacologic intervention (paroxetine) improved fatigue
Yang et al., 2017 [32]	Meta-analysis of RTCs, *n* = 11 trials (723 patients)		-	MFIS, FSS	Amantadine, not modafinil, was effective for treating the fatigue in MS. Current data could not answer whether treatment of MS fatigue using aspirin or 4-AP was beneficial.
Nourbaksh et al., 2017–2019 [33]	Randomized, controlled trial, multicenter study, *n* = 132	Methyl-phenidate, modafinil, and amantadine	Placebo	MFIS	No results posted.
Triche et al., 2016 [34]	Observation-al pre–post study, *n* = 39, 14 weeks	Dalfampridine	No control group	PS	After drug treatment for 14 weeks, a significant improvement in the SDMT (*p* < 0.001) and the PS Fatigue score (*p* = 0.04). Timed walk responders had significant improvements in SDMT (*p* < 0.001) and PS fatigue (*p* = 0.046) from baseline to week 14.

Abbreviations: 4-AP: 4-aminopiridine; FSS: Fatigue Severity Scale; EDSS: Expanded Disability Status Scale; ALCAR: Acetyl-l-carnitine; MFIS: Modified Fatigue Impact Scale; MS-FS: MS-Specific Fatigue Scale; SDMT: Symbol Digit Modalities Test; PASAT: Paced Auditory Serial Addition Test; PS: Performance Scales.

**Table 2:** Different types of physical activity performed by multiple sclerosis (MS) patients with fatigue.

Study, Year, PEDro Score, Reference	Study Design	Type of Intervention	Outcome Measures	Main Findings
Hasanpour et al., 2016 PEDro: 5/10 [45]	Randomized, controlled trial; *n* = 90	**Yoga, aerobics exercises:** 12 weeks, 3 sessions per week, 40 min per session	Rotten fatigue test, SF-36	Fatigue decreased in yoga and exercise groups, but in the control group, the fatigue severity increased. Physical function, physical and emotional role, social function, energy, mental status and overall hygiene increased; pain and fatigue were relieved among patients.
Mokhtarzade et al., 2017 PEDro: 5/10 [46]	Randomized, controlled trial; *n* = 40	**Aerobic exercise:** 8 weeks, 3 days per week, upper and lower limb aerobic interval training program	FSS, MSQOL-54	Significant decrease in fatigue after the 8-week aerobic interval training (*p* < 0.05). A considerable change in MSQOL-54 (total) and physical and mental quality of life subsequent to the exercise training (*p* < 0.05).
Mostert S, et al., 2002 PEDro: 3/10 [47]	Clinical trial; *n* = 26	**Aerobics exercise:** 4 weeks, 5 sessions a week, 30 min per session, bicycle exercise with individualized intensity	FSS, SF-36	A significant rightward placement of the aerobic threshold (VO2 + 13%; work rate + 11%), an improvement of health perception (vitality + 46%; social interaction + 36%), an increase of activity level (+17%) and a tendency to lower fatigue in the MS training group. The level of excessive fatigue measured by FSS was 60–67% higher in MS groups in comparison to matched controls.
Devasahayam et al., 2020 PEDro: none [48]	Clinical trial; *n* = 10	Aerobic walking training in a room cooled to 16 °C using bodyweight-supported treadmill	FSS, MFIS, SF-36	Fatigue in MFIS significantly improved. The effect was sustained after 3 months.
Kargarfard et al., 2017 PEDro: 7/10 [49]	Randomized, controlled trial; *n* = 34	**Aquatic exercise:** 8 weeks, 3 sessions per week, sessions 45–60 min, water temperature: 28–30 °C	MFIS	Aquatic exercise training improved functional capacity, balance, and perceptions of fatigue in women with MS. All outcome measures improved in the experimental group: MFIS (pretest mean ± SD, 43.1 ± 14.6; post-test mean ± SD, 32.8 ± 5.9; *p* < 0.01).
Kooshiar et al., 2015 PEDro: 4/10 [50]	Randomized, controlled trial; *n* = 37	**Aquatic therapy:** 8 weeks, 3 sessions per week and 45 min per session, water temperature: 28–29.5 °C	FSS, MFIS, MQLIM	Significant effects of aquatic exercise on physical and psychosocial fatigue perception, QoL, and fatigue severity (*p* = 0.001). Non-significant effect for cognitive fatigue perception (*p* > 0.05).
Razazian, et al., 2016 PEDro: 6/10 [51]	Randomized, controlled trial; *n* = 54	**Aquatic exercise:** 8 weeks, 3 sessions per week and 1h per session, water temperature: 28–30 °C **Yoga:** 8 weeks, three times per week, about 60 min	FSS, Beck Depression Inventory, 10-point visual analogue scale for paresthesia	A significant decrease in the yoga and aquatic exercise groups compared with the non-exercise control condition and fatigue, depression, and paresthesia over time.
Garrett et al., 2013 PEDro: 6/10 [52]	Randomized, controlled trial	**Physiotherapist (PT)-led exercise** (*n* = 80), **yoga** (*n* = 77), **fitness instructor (FI)-led exercise** (*n* = 86)	MFIS, MSIS	Statistically significant improvement in the MSIS-29 psychological component and both the MFIS total and physical subscales, which were greater than the control (*p* < 0.05).
Tarakci et al., 2013 PEDro: 8/10 [56]	Randomized, controlled trial; *n* = 99	**Group exercise led by physical therapist**	FSS	Statistically significant improvements for all outcome measures in the exercise group (*n* = 51) (*p* < 0.01). In the control group (*n* = 48), there was a statistically significant increment only in the FSS score (*p* = 0.002).
Sangelaji et al., 2014 PEDro: 3/10 [57]	Randomized, controlled trial; *n* = 59	**Combination exercises:** 10 weeks, 3 sessions a week, 20–40 min per session, stretching and aerobic exercises, strengthening exercises with and balancing exercises.	FSS, 6-min Walk Test, EDSS quality of life tests	Significant changes in the intervention group in comparison to the control group in the second phase of the study compared to the first one for all tests except EDSS, *p* = 0.60 (EDSS mean values at the beginning: intervention group: 1.7; control group 1.96; at the end: intervention group: 2.2; control group: 2.74); FSS: −6.9, *p* = 0.02, Mental Quality of Life: 16.36, *p* = 0.001; Physical QOL: 12.17, *p* = 0.001, six minute walking: 137.2, *p* < 0.0001; Berg: 3.34, *p* < 0.0001.
McCullagh et al., 2008 PEDro: 4/10 [58]	Randomized, controlled trial; *n* = 30	**Exercise:** 3 months, 2 sessions per week, participants also exercised independently once a week.	MFIS, MSIS-29, FAMS	Exercise group had significantly greater improvements in exercise capacity and fatigue (MFIS: -13 in exercise group versus 1 in control group, *p* = 0.02). Improvements in QOL and fatigue lasting beyond the program.

Abbreviations: PEDro: Physiotherapy Evidence Database; FSS: Fatigue Severity Scale; MFIS: Modified Fatigue Impact Scale; MQLIM: Multicultural Quality of Life Index; QoL: quality of life; MSIS: Multiple Sclerosis Impact Scale; MSQOL-54: Multiple Sclerosis Quality of Life Questionnaire; SF-36: 36-Item Short Form Health Survey; MSIS-29: Multiple Sclerosis Impact Scale-29; FAMS: Functional Assessment of Multiple Sclerosis.

**Table 3:** Clinical studies of cold therapies in patients with MS-related fatigue.

Study, Year, PEDro Score, Reference	Study Design	Potential Intervention	Outcome Measures	Main Findings
Miller et al., 2016 PEDro: none [61]	Case–control study; *n* = 24	10 × 3 min WBC sessions (one exposure per day at −110 °C or lower)	FSS, RMA, MSIS-29, EDSS	Improvement in the functional status and in the feeling of fatigue. High fatigue group achieved better results than low fatigue, especially in the MSIS-29-PHYS, MSIS-29-PSYCH, RMA1, and RMA3. Outcomes in the EDSS, RMA2, and FSS were similar in both groups. Mean EDSS in low fatigue group before treatment: 5.1 ± 0.7, after: 4.8 ± 0.7; Mean EDSS in high fatigue group before treatment: 5.2 ± 1.1, after: 5.0 ± 1.1
Gonzales et al., 2017 PEDro: 4/10 [62]	Randomized, controlled trial; *n* = 18	7-week physical training program with a cooling vest during each training session	SEP-59	Emotional well-being and cognitive functions investigated in SEP-59 were significantly improved (*p* < 0.05), and general and physical fatigue significantly decreased (*p* < 0.05).
Özkan et al., 2017 PEDro: none [63]	Case–control study; *n* = 75	Colling suit (vest) applied once a day for 40 min, 4 weeks	FIS, FSS, and Modified Barthel Index.	Improvements from baseline in all measures of fatigue At the 4th-week measurement, the experimental group scored significantly better on the Modified Barthel Index
Nilsagård et al., 2006 PEDro: 7/10 [64]	Randomized, controlled crossover study; *n* = 43	Single session with Rehband cooling garment	A study-specific questionnaire to evaluate subjective experiences. 10TW, 30TW, TUG, oral temperature, spasticity, standing balance	Improvement in 10TW, 30TW, one-legged stance, tandem stance (right) and TUG. Improvements in fatigue, spasticity, weakness, balance, gait, transfers, ability to think clearly and time to recover.

Abbreviations: PEDro: Physiotherapy Evidence Database; WBC: Whole-body cryostiumlation; FSS: Fatigue Severity Scale; EDSS: Expanded Disability Status Scale; RMA: Rivermead Motor Assessment; MSIS-29: Multiple Sclerosis Impact Scale; SEP-59: French version of the Multiple Sclerosis Quality Of Life; FIS: Fatigue Impact Scale; 10TW: 10-metre timed walk; 30TW: 30-metre timed walk; TUG: timed “up and go”.

**Table 4:** Clinical studies of magnetic field therapy in patients with MS-related fatigue.

Study, Year, PEDro Score, Reference	Study Design	Potential Intervention	Outcome Measures	Main Findings
Lappin et al., 2003 PEDro: 7/10 [65]	Multi-site, double-blind, placebo-controlled, crossover trial; *n* = 117	Daily exposure to a small, portable PMFT generator	MSQLI	Improvements in fatigue and overall quality of life were significantly greater in the active device group.
Piatkowski et al., 2009 PEDro: 7/10 [66]	Randomized, double-blind, controlled trial; *n* = 37	BEMER magnetic field treatment for 8 min twice daily in comparison to placebo for 12 weeks	MFIS, FSS	A significant difference of MFIS value after 12 weeks in favor of the verum group (26.84 versus 36.67; p 1⁄4 0.024). FSS values were significantly lower in the verum group after 12 weeks (3.5 versus 4.7; p 1⁄4 0.016). After 6 weeks follow-up, the groups did not differ in fatigue (MFIS, FSS). MFIS: a significant decrease in physical (p1⁄40.018) and cognitive (p1⁄40.041), but not in psychologic subscales; only in the verum group regarding the baseline and 12 week timepoints.
De Carvalho et al., 2012 PEDro: 6/10 [67]	Randomized, double-blind, crossover trial; *n* = 50	Systemic pulsed low-frequency magnetic field with an intensity of 37.5 mT and with a sequence of pulses at 4–7 Hz. Total of 24 sessions, three times a week for 8 weeks, 24 min per session	FSS, MFIS,	Improvement in MFIS Physical Score for T0 (beginning of treatment) −T1 (end of treatment) (*p* < 0.05) for time but not for treatment and time × treatment factors.
Mostert et al., 2005 PEDro: 6/10 [68]	Randomized, controlled trial; *n* = 25	PMFT, single treatment lasted 16 min twice daily over 3–4 weeks	FSS, VAS	Over time of rehabilitation, fatigue was reduced by 18% in TG and 7% in CG, which was not statistically significant. A statistically significant immediate effect of the single treatment session with 18% reduction of fatigue (in VAS) in treatment group versus 11% in control group

Abbreviations: PEDro: Physiotherapy Evidence Database; FSS: Fatigue Severity Scale; MFIS: Modified Fatigue Impact Scale; EDSS: Expanded Disability Status Scale; BDE: Beck Depression Inventory; FAMS: Functional Assessment of Multiple Sclerosis; PMFT: Pulsed Magnetic Field Therapy; MSQLI: Multiple Sclerosis Quality of Life Inventory; VAS: Visual Analog Scale.

**Table 5:** Clinical studies of FES in patients with MS-related fatigue.

Study, Year, References	Study Design	Potential Intervention	Outcome Measures	Main Findings
Chang et al., 2011 [70]	*n* = 9	8 weeks of quadriceps muscle surface FES training	Maximal voluntary contraction, voluntary activation level, twitch force, FI, CFI, Peripheral Fatigue Index, and MFIS	FI (*p* = 0.01), CFI (*p* = 0.02), and MFIS (*p* = 0.02) scores improved significantly Improvements in central fatigue contributed significantly to improvements in general fatigue (*p* < 0.01).
Pilutti et al., 2019 [71]	Randomized, controlled trial, *n* = 11	FES cycling exercise (*n* = 6) or passive leg cycling (*n* = 5) for 24 weeks	FSS, MFIS, SF-PMQ, MSIS-29	Moderate to large improvements in cognitive processing speed (d = 0.53), fatigue severity (d = −0.92), fatigue impact (d = −0.45 to −0.68) and pain symptoms (d = −0.67)

Abbreviations: FSS: Fatigue Severity Scale; MFIS: Modified Fatigue Impact Scale, SF-PMQ: Short-Form McGill Pain Questionnaire; MSIS-29: 29-Item Multiple Sclerosis Impact Scale; FI: General Fatigue Index; CFI: Central Fatigue Index.

**Table 6:** Clinical studies of non-invasive brain stimulation in patients with MS-related fatigue.

Study, Year	Study Design	Type of Intervention	Outcome Measures	Main Findings
Chalah et al., 2020 [73]	randomized, sham-controlled study, *n* = 11	bilateral tDCS	FSS, MFIS	Active but not sham tDCS resulted in a significant improvement of fatigue at day 5 (*p* < 0.05), an effect that seems to last at least 1 week following the stimulation (*p* = 0.05).
Cancelli et al., 2018 [74]	randomized, double-blind, sham-controlled, crossover study, *n* = 10	tDCS	MFIS	The amelioration of fatigue symptoms after real stimulation (40% of baseline) was significantly larger than after sham stimulation (14%, *p* = 0.012). Anodal whole-body S1 induced a significant fatigue reduction in mildly disabled MS patients when the fatigue-related symptoms severely hampered their quality of life.
Tecchio et al., 2014 [75]	randomized, double-blind, sham-controlled, crossover study, *n* = 10	anodal bilateral primary somatosensory areas tDCS	MFIS	The real neuromodulation by a personalized electrode reduced fatigue in all patients by 26% on average (*p* = 0.002), which did not change after sham (*p* = 0.901).
Saiote et al., 2014 [76]	sham-controlled, double-blind intervention study	excitability-enhancing anodal tDCS	FSS, MSFSS, MFIS	In the whole group, the analysis scores of the fatigue scales were not altered by tDCS. In an exploratory analysis, a correlation between response to the stimulation regarding subjectively perceived fatigue and lesion load in the left frontal cortex was tested. Patients responding positively to anodal tDCS had higher lesion load compared to non-responding patients.

Abbreviations: tDCS: Transcranial Direct Current Stimulation; FSS: Fatigue Severity Scale; MFIS: Modified Fatigue Impact Scale; MSFSS: Multiple Sclerosis Specific Fatigue Severity Scale.
